# Evidence-based strategies needed to combat malnutrition in Sub-Saharan countries facing different stages of nutrition transition

**DOI:** 10.1017/S1368980021001221

**Published:** 2021-08

**Authors:** Hélène Delisle, Mieke Faber, Pascal Revault

**Affiliations:** 1 Department of Nutrition, Faculty of Medicine, University of Montreal; 2 Non-Communicable Diseases Research Unit, South African Medical Research Council; 3 Action contre la Faim (ACF), France

No continent or country is exempt from malnutrition, which may take several forms that may even be combined: undernutrition, micronutrient deficiencies, obesity and other diet-related chronic diseases and risk thereof. Some populations are more affected than others primarily owing to poverty, and Sub-Saharan Africa (SSA) is among those. However, SSA, which includes forty-eight countries, is not a monolith, with low- and as well as middle-income countries, and even two high-income country (Mauritius and Seychelles). Out of twenty-nine low-income countries in 2019 (GNI < $1036), twenty-three are in SSA, while only twenty-one out of the 106 middle-income countries ($1036 – $12 535) are in SSA^(1)^. It is therefore not surprising that the burden of malnutrition is particularly heavy in SSA, with 32·7 % of children under five being stunted in 2019^(2)^. Also, many SSA countries face the double burden of malnutrition^(3)^, that is, the overlap of undernutrition and overweight/obesity in the same population, or even the triple burden of malnutrition (stunting in children under five, anaemia in women of reproductive age and overweight/obesity in adult women). Of forty-one countries that faced the triple burden of malnutrition in 2018, thirty were in Africa^(4)^.

This special issue of PHN on maternal and child nutrition in SSA is particularly relevant given the nutrition challenges facing the region and the vulnerability of mothers and young children to malnutrition. Nutrition interventions worldwide focus on the ‘First 1000 days’ as a critical window of opportunity to intervene. Preconception nutrition is also increasingly recognised as being very important^(5)^. This special issue is a reminder that research is essential for achieving progress in tackling all forms of malnutrition even in resource-poor regions such as SSA, and beyond the 1000 d. It is also timely in consideration of the current global mobilisation for improved nutrition. However, the call for papers predated the COVID-19 pandemic. This crisis is not addressed in the papers, although it severely affects food security and nutrition in SSA as well as in other fragile regions^(6)^.

Nutrition has been enjoying a particular momentum recently. The need to improve nutrition globally is recognised in the Sustainable Development Goals adopted by the UN in 2015, with two of the seventeen Sustainable Development Goals explicitly targeting nutrition^(7)^. Sustainable Development Goal 2 aims to end hunger, achieve food security and promote sustainable agriculture, and Sustainable Development Goal 3 is to ensure healthy lives and promote well-being for all at all ages which includes enabling people to make healthy food choices. Another major milestone is the Decade of Action on Nutrition (2016–2025), which is a commitment by the UN and member countries to undertake 10 years of sustained and coherent implementation of policies, programmes and increased investments to eliminate malnutrition in all its forms, everywhere, leaving no one behind^(8)^. The WHO’s Member States have endorsed global nutrition targets for 2025, which include substantial reductions of stunting, anaemia, low birth weight and wasting while increasing the rate of exclusive breast-feeding in the first 6 months and avoiding any increase in childhood overweight. The Lancet Journal is increasingly involved in nutrition, and in 2019 only, two important Commission Reports pertaining to nutrition were published, one on the global syndemic of obesity, undernutrition and climate change^(9)^ and a second one on healthy diets for sustainable development^(10)^. The same year, The Lancet also published the WHO Series on the double burden of malnutrition^(11)^. Yet, one may wonder why progress has so far been so meagre despite the opportunities offered by these goals and commitments intended to achieve nutrition impact. Acknowledging that progress on malnutrition is not only too slow but also unfair, the 2020 Global Nutrition Report introduces the concept of nutrition equity, identifies priority actions focusing on food and health systems and stresses the need for better nutrition coordination, financing and accountability^(12)^.

The papers published in this special issue report on innovative studies, qualitative or quantitative, in SSA, with the focus on optimal nutrition in children, adolescents and women of reproductive age (pre-pregnant, pregnant and lactating). The special issue includes a total of nineteen papers. Nearly all papers come from English-speaking countries, the only exception being French-speaking Burkina Faso (three papers). Interestingly, ten papers report on qualitative studies, whereas publications using quantitative research are usually predominant. Four qualitative studies looked at community solutions to malnutrition in a novel and participatory approach with the population^(13–16)^, while one study looked at factors influencing the implementation of a multisectoral, community-level nutrition programme aimed at improving infant and young child feeding practices^(17)^. The other qualitative studies explored nutrition-related behaviours of mothers^(18,19)^, women outside of their maternal role^(20)^, parents^(21)^, and adolescents^(22)^.

According to the ecological framework, food choices are shaped by individual factors, as well as social, physical and macro-level environments^(23)^. Poverty and concerns related to affordability of food were reported to influence food choices in most of the studies included in this special issue. The nutrition transition currently observed in low- and middle-income countries, including African countries, is driven to a large extent by changes in local food systems^(3)^. SSA countries are however at different stages of economic and nutrition transition. Four papers report on the community’s perceptions of factors affecting maternal and child nutrition in three SSA countries that are at different stages of transition, namely Burkina Faso, Ghana and South Africa, and results show that food choices and barriers to optimum nutrition reflect the stage of the country’s transition^(13–16)^. In Ghana, for example, lack of irrigated agricultural land and poor harvests were reported as main barriers to optimal nutrition^(13)^. In contrast, in the urban setting in South Africa, which has a more modernised food system, there is an increasing reliance on processed takeaway foods^(14)^. In Ethiopia, concerns related to hygiene in the physical food environment and informal food outlets were key factors influencing the food choices of adolescents, and as a result, packaged foods were viewed as healthier options^(22)^.

Qualitative studies further highlight the importance of family dynamics, cultural beliefs and social media with regard to food choices. Infant feeding is reported to be strongly influenced by family matriarchs in South Africa, although the advice given is not aligned with infant and young child feeding recommendations^(18)^. The conflict between traditional knowledge on infant feeding and information provided by health workers is also highlighted in a study in Rwanda^(19)^. The importance of social media as source of nutrition information and determinant of food choices is reflected in a study in Ghana^(20)^. In a study in South Africa, parents noted that preschool children even at this very young age are drawn to devices such as smartphones, tablets and laptops and that TV was regularly watched; these habits may limit physical activity, thereby favouring overweight^(21)^.

Out of the nine quantitative papers in this special issue, seven are epidemiological studies and only three pertain to interventions. The epidemiological studies focused on iodine^(24,25)^, anaemia^(26,27)^, linear growth^(28)^ and obesity^(29)^. All three intervention studies deal with undernutrition^(30–32)^. The double burden of malnutrition and the ‘double duty actions’^(33)^ are not specifically addressed although the issue is discussed in the qualitative papers on community solutions.

None of the studies included in this issue applied implementation research methods and tools even if one study examined the facilitating factors and challenges of implementing multisectoral nutrition programmes^(17)^. This is an area where further research should be advocated. It is also unfortunate that no selected paper dealt directly with local food systems considering their importance for nutritional health and sustainability of the planet. Globally, SSA is the region with the highest prevalence of hunger (22·0 % in 2019), and food security in the region is adversely affected by a wide range of factors including poverty, conflicts, violence and changes in environmental conditions^(34)^. At the same time, changes in the food systems have resulted in less-nutritious food being cheaper, more accessible and more convenient which, together with reduced physical activity, have led to increasing overweight and obesity^(35)^. Food systems in SSA therefore need to supply affordable and environmentally sustainable healthy diets to both the undernourished and the overweight.

It is globally recognised that nutrition strategies on their own will not be sufficient to eliminate malnutrition. Combining nutrition-specific interventions such as breast-feeding promotion or micronutrient fortification and nutrition-sensitive interventions addressing underlying causes of malnutrition (such as food insecurity or poor sanitation) is needed^(36)^. These interventions however need to be context-specific, considering local factors as highlighted in several of the papers in this issue.

The annual research conference on undernutrition organised by ACF/Action Against Hunger is of importance for advancing evidence-based strategies to combat under nutrition in low- and middle-income countries, and notably in SSA^(37)^. It is intended to share research findings with the humanitarian, developmental and academic networks. The conclusions of the last conference held in December 2019 corroborate some of the recommendations arising from the work presented in this special issue^(38)^. In particular: the effectiveness of concrete actions that involve families and individuals within the community to tackle stunting; the need to also address food systems; and the relevance of revisiting more broadly the determinants of under nutrition such as those related to gender. The importance of considering undernutrition as a continuum, with the co-existence of wasting, stunting and micronutrient malnutrition, integrating prevention and treatment, and based on intervention research was stressed by all participants.

This special issue on maternal and child nutrition in Africa fostered the publication of African researchers’ work. It is to be hoped that another special issue on nutrition in Africa will be programmed in the near future, this time with emphasis on the growing issue of the double burden of malnutrition and on diet-related non-communicable diseases.
